# Innovation to impact in spatial epidemiology

**DOI:** 10.1186/s12916-018-1205-5

**Published:** 2018-11-14

**Authors:** Andrew J. Tatem

**Affiliations:** WorldPop, Department of Geography and Environment, University of Southampton, Highfield, Southampton, SO17 1BJ UK

**Keywords:** Disease mapping, spatial scales, policy, implementation

## Abstract

Spatial epidemiology is a rapidly advancing field, pushing our abilities to measure, monitor and map pathogens at increasingly finer spatiotemporal scales. However, these scales often do not align with the abilities of control programmes to act at them, building a disconnect between academia and implementation. Efforts are being made to feed innovations into government, build spatial data skills, and strengthen links between disease control programmes and universities, yet work remains to be done if goals for disease control, elimination and ‘leaving no one behind’ are to be met.

## Background

The past couple of decades has seen spatial epidemiology become a major area of activity [1]. Driven by factors such as the increasing use of global positioning systems in disease prevalence surveys, health facility mapping and the collection of genetic data, spatially precise epidemiological data are now becoming more widespread. Additionally, spatial epidemiology continues to be a rapidly growing field, with the mapping of health data, the rising availability of epidemiologically relevant variables from satellites and the continued development of statistical methods. Furthermore, the development of rapid diagnostic tests, the use of mobile phone-based reporting, and the advancement of genome sequencing technology add a new dimension in terms of increases in the frequency and speed of data collection. A feature of the above advancements has been a rising trend within the academic world in the ability to map and understand disease patterns and dynamics at increasingly finer spatial and temporal scales, launching new terms such as ‘precision public health’ [2]. Where once ‘subnational disease mapping’ meant moving from national to provincial scales, we can now rapidly uncover household-level patterns in the genetic makeup of pathogens [3] and predict, with some accuracy, the distributions of diseases and their vectors at a resolution of kilometres across the globe [4].

Every time we sharpen the lens of data collection and analyses to increasingly finer scales we see substantial heterogeneity. For example, in the case of malaria, the number of annual cases may be counted at district level, with some areas having a far greater number of cases than others. Taking a closer look at the district with the most cases, certain settlements within it are seen to contribute most of the cases [5]. Subsequent mapping within these settlements highlights neighbourhoods with a greater number of cases [6] and, within these, neighbouring households with significantly different case loads [7]. Thus, the message put forward in many spatial epidemiology studies is that through our improved understanding, mapping and analysis at increasingly finer scales, highlighting ‘hotspots’ [8] or ‘coldspots’ [9], we can highlight communities left behind, pinpoint cases, measure risks and map transmission in a much more effective, efficient and cost-saving way, facilitating the improved targeting of limited resources.

The potential of these new data streams and methods is substantial, providing tools and insights to tackle diseases across types, endemicity spectra and spatiotemporal scales, and tailoring approaches to needs, whether to stratify risk for endemic diseases or to track down the last few cases to eliminate a pathogen. However, this all needs to be balanced against the ability of over-burdened and under-resourced disease control programmes to adopt new methods and act at such fine scales. A national disease control programme that plans, operates and implements at district levels faces major challenges in using and acting on finer scale insights. Additionally, research is often lacking on what scales increase noise, obscure patterns and become cost-prohibitive for action. Geographical Information Systems are becoming increasingly widespread tools for monitoring, mapping and analysing disease data; however, the costs of licenses and training can be prohibitive in some settings. Moreover, while academia races ahead in developing new methods, insights and outputs, these can be slow to filter through to those tasked with controlling and eliminating the diseases. The low uptake and use of risk maps, modelling and ‘big data’ in national decision-making is evidence of this [10, 11]. Such a lag has and always will exist; yet, if ambitious global and national goals for disease control, elimination and ‘leaving no one behind’ are to be met, there is a need to start closing the gap.

The disconnect between academia and health ministries is often a limiting factor in the uptake of new methods, yet disconnects between academic fields and within government can also present obstacles. Valuable research and investments in precise diagnostics, vaccination tracking and surveillance systems can be undermined by a lack of similar efforts in measuring and mapping populations to provide the important context of denominators at small spatial scales – are the 100 cases of disease ‘X’ identified coming from a population of 1000 or 10,000? The phenomenon of vaccination coverage rates of greater than 100% is common through the use of outdated census counts as denominators [12, 13], while national disease surveys struggle to be representative if reliant on sample frames that are many years old in countries with rapidly changing populations [14].

## Conclusions

The lags in government adoption of scientific innovations in spatial epidemiology as well as disconnects between academia and disease control programmes are being tackled, with many positive examples that point towards recipes for bridging gaps. Links between ministries of health and local universities are being strengthened, training in Geographical Information Systems is becoming more widespread together with the adoption of open-source or tailored systems and dashboards [15], and organisations and funding programmes are focussing on feeding innovations into health systems and national statistics offices, sometimes requiring that modellers be embedded in ministries of health. Further, major funders and implementing partners are increasingly building explicitly applied public health goals into funding proposals; these require ministries of health to be involved and success is judged on uptake and implementation of new methods (e.g. [16]). The problems of inaccurate or missing denominators are being addressed through the integration of survey, satellite and mobile phone data, feeding into dashboards and tracking systems used by ministries of health [17]. While these efforts are gaining traction, they are not quite yet the norm, with academics often still rewarded more for quick-win publications in top journals than for the slower and more political process of engagement in the development of new insights and methods with national programmes to ensure sustained adoption and impact. On the implementation side, workloads and financial constraints often leave no time for learning about new findings and approaches.

These are exciting times for spatial epidemiology research, as the integration of multiple types of spatially referenced data within powerful new methods pushes us to new frontiers of spatial and temporal precision with great potential for impact. However, unless these are built considering the needs, limitations and capacity of those tasked with designing strategies and acting upon them, and unless appropriate incentives exist to facilitate this, the danger is that such work remains as pretty maps in scientific journals, circulating only in academic bubbles.
